# Jejunogastric intussusception after Whipple procedure with B-II reconstruction: a case report

**DOI:** 10.1186/s12876-020-01259-2

**Published:** 2020-04-10

**Authors:** Yun-Xiao Lyu, Yue-Ming Xu

**Affiliations:** 1 Department of Hepatobiliary Surgery, Dongyang People’s Hospital, No. 60, West Wuning Road, Dongyang, Jinhua, Zhejiang China; 2 Department of General Surgery, DongyangPeople’s Hospital, No. 60, West Wuning Road, Dongyang, 322100 Zhejiang Province China

**Keywords:** Jejunogastricintussuception, Whipple, Complication

## Abstract

**Background:**

Jejunogastric intussusception (JGI) is a rare but severe complication after gastric surgery. JGI can occur from a few days to 55 years postoperatively and has a reported incidence of < 0.1% in patients who undergo gastric surgery. We firstly report a male patient with duodenal cancer who underwent Whipple’s procedure with side-to-side gastrojejunostomy and who subsequently developed JGI. A literature review is provided.

**Case presentation:**

A 68-year-old man was admitted to our emergency department with left upper quadrant abdominal pain and hematemesis of 4 h’ duration. He had undergone Whipple’s procedure (duct-to-mucosa pancreaticojejunostomy and side-to-side gastrojejunostomy) with B-II reconstruction for duodenal papillary adenocarcinoma 5 years earlier. His vital signs were stable with a blood pressure of 163/93 mmHg, temperature of 37.0 °C; and heart and respiratory rates of 86 per/min and 20 per/min, respectively. Physical assessment showed mild tenderness in the left upper quadrant, only. A complete blood count showed white cell and platelet counts of 11.69 × 10^3^/L and 196 × 10^3^/L, respectively, and a hemoglobin level of 13.5 g/L. Abdominal computed tomography (CT) suggested a retrograde intussusception of the intestines into the stomach with dilatation of the remnant stomach. The patient immediately underwent exploratory laparotomy, which revealed a 20-cm retrograde efferent limb at the remnant stomach that had travelled through the previous gastrojejunostomy. There was no evidence of malignancy. We manually reduced the intussuscepted loop using gentle traction, and the viability of the intestinal loop was preserved. The patient had an uneventful postoperative recovery.

**Conclusion:**

JGI is a rare but potentially fatal complication after gastric surgery, especially following Whipple’s procedure. Early diagnosis and treatment are crucial, and surgery is considered the most effective treatment for JGI.

## Background

Jejunogastric intussusception (JGI) is a rare but severe complication after gastric surgery. JGI can occur from a few days to 55 years postoperatively and has a reported incidence of < 0.1% in patients who undergo gastric surgery [1–3]. The clinical presentations of JGI vary; however, surgery is the standard treatment, and endoscopy has been used in a small number of patients [4, 5]. Fewer than 200 cases of postoperative JGI have been reported. We report a male patient with duodenal cancer who underwent Whipple’s procedure with side-to-side gastrojejunostomy and who subsequently developed JGI.

## Case presentation

**Fig. 1:**
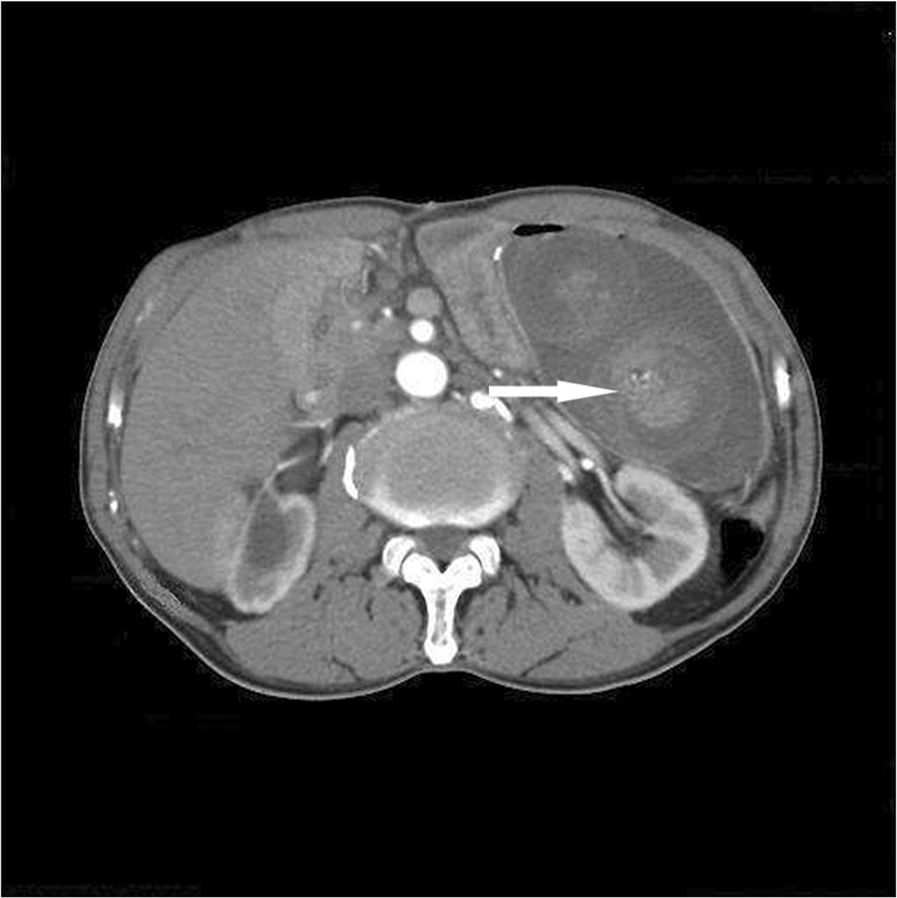
Abdominal CT scan. The CT-scan reveals dilated stomach with bowel loops (Arrow: jejunogastric intussusception)

**Fig. 2:**
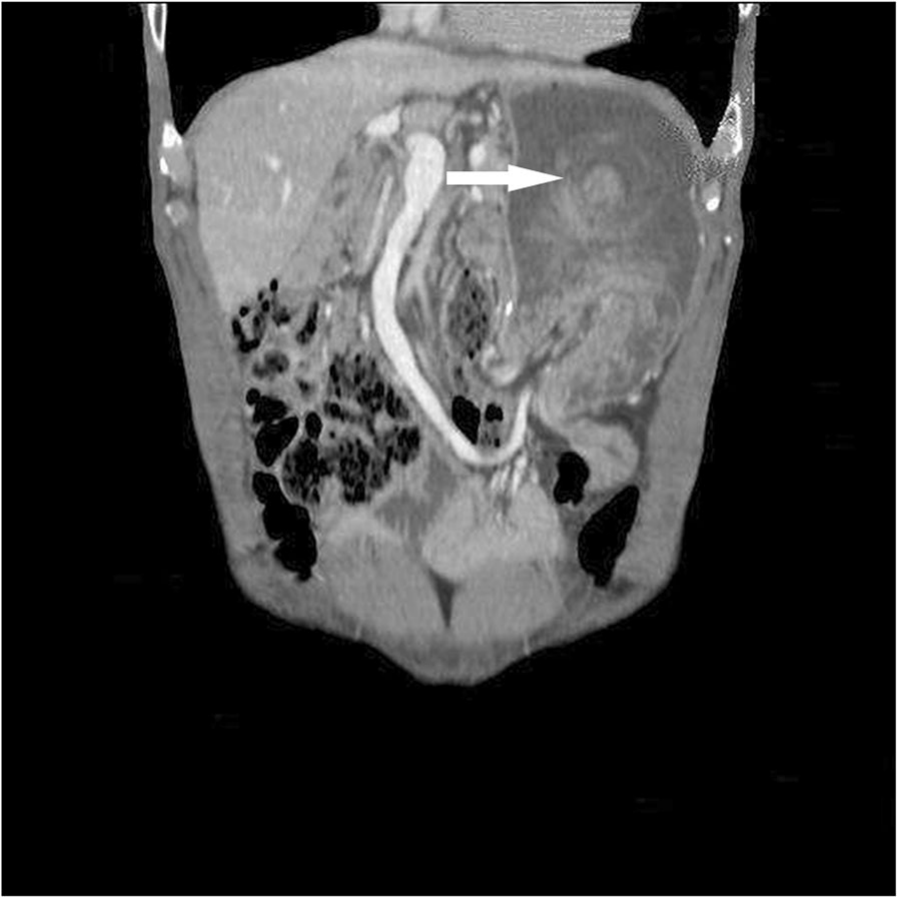
Abdominal CT scan. Another section showed loop of jejunum in the body of stomach (Arrow: jejunogastric intussusception)

**Fig. 3:**
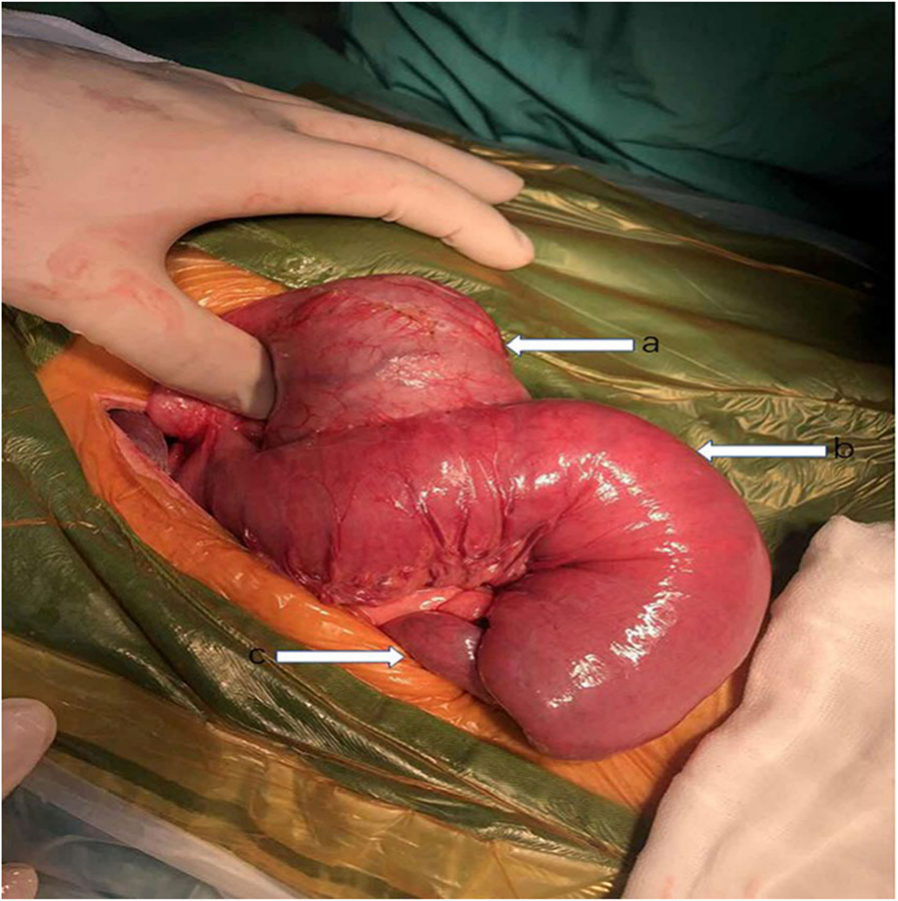
Operative view demonstrating JGI. Arrow a showed remnant stomach; Arrow b indicates intussusceptum. Arrow C indicates intussusceptien

A 68-year-old man was admitted to our emergency department with left upper quadrant abdominal pain and hematemesis of 4 h duration. He had undergone Whipple’s procedure (duct-to-mucosa pancreaticojejunostomy and side-to-side gastrojejunostomy) with B-II reconstruction for duodenal papillary adenocarcinoma 5 years earlier. His vital signs were stable with a blood pressure of 163/93 mmHg, temperature of 37.0 °C; and heart and respiratory rates of 86 per/min and 20 per/min, respectively. Physical assessment showed mild tenderness in the left upper quadrant, only. A complete blood count showed white cell and platelet counts of 11.69 × 10^3^/L and 196 × 10^3^/L, respectively, and a hemoglobin level of 13.5 g/L. Abdominal computed tomography (CT) suggested a retrograde intussusception of the intestines into the stomach with dilatation of the remnant stomach (Figs. 1 and 2). The patient immediately underwent exploratory laparotomy, which revealed a 20-cm retrograde efferent limb at the remnant stomach that had travelled through the previous gastrojejunostomy (Fig. 3). There was no evidence of malignancy. We manually reduced the intussuscepted loop using gentle traction, and the viability of the intestinal loop was preserved. The patient had an uneventful postoperative recovery.

## Discussion and conclusions

Jejunogastric intussusception (JGI) is a rare complication after gastric surgery and was first reported in 1914 by Bozzi [6]. JGI can occur at any gastric anastomosis including following gastrojejunal anastomosis and Braun’s anastomosis in Billroth II reconstruction, and at the Y anastomosis site in Roux-en-Y reconstruction [7]. The primary disease prior to gastric surgery can include gastric cancer or gastric ulcer [8–13]; however, with increasing numbers of bariatric surgeries, the incidence of JGI is also increasing [14]. To our knowledge, ours is the first report of JGI after Whipple’s procedure.

JGI can have varied clinical presentations such as abdominal pain, vomiting, intestinal obstruction, and hematemesis, which appear acutely. Approximately 50% of patients can also present with an abdominal mass [15–17]. Previous studies discussed a chronic form of JGI, which may mimic the acute form, but signs are milder, transient, and resolve spontaneously [18]. These clinical presentations may help diagnose JGI, which is crucial. Methods of diagnosis include gastroendoscopy, abdominal ultrasonography, and CT. Gastroscopy can provide immediate images and a diagnosis of JGI; however, gastroscopy is not appropriate in patients with peritonitis. Abdominal ultrasonography is widely used in emergency departments but cannot be used to diagnosis JGI because of profuse intestinal and gastric gas. Computed tomography can also provide immediate images of abdominal pathological findings [19], and may be the most useful method because CT can also evaluate the viability of the intestine.

According to Shackman’s classification, JGI is classified into three types: type I: afferent loop intussusception; type II: efferent loop intussusception; and type III: both loops are involved in the intussusception [2]; efferent loop intussusceptions occur in 70% of patients with JGI [20]. Brynitz and Rubinstein suggested a more detailed approach: type1: afferent loop intussusception; type 2a: efferent loop intussusception; type 2b: efferent–efferent loop intussusception; type 3: combined types 1 and 3; and type 4: intussusception through Braun’s side in a jejunojejunal anastomosis [21]. Our patient fulfilled the criteria for type II or type 2a according to Brynitz and Rubinstein’s classification.

The etiology of JGI is unclear, and several possible mechanisms have been proposed, namely, a long afferent or efferent loop, high acidic state, increased intra-abdominal pressure, and shortening of the jejunal mesentery [22]. In terms of surgery, the technique of digestive tract reconstruction, such as the size of gastrojejunostomy, the length of afferent or efferent loop, may play important role in the formation of JGI. however, none of these mechanisms have been confirmed. In some patients, retrograde peristalsis was considered the cause of type II JGI [3, 22]. Additionally, the laxity of mesentery may be related to the JGI.

Early treatment for JGI is crucial because of the high risk of incarceration and strangulation; delayed treatment can lead to serious consequences and even death. Prompt surgery remains the most important treatment and should be considered in every patient with JGI. Surgical procedures for JGI include reduction, resection, and reconstruction of the anastomosis. The mortality rate is linked to operation time; previous studies reported a mortality of approximately 50% with operation times > 48 h compared with 10% for operation times < 48 h [23]. The choice of surgical procedure depends on the operative findings, and the most important point is judging the viability of the intussusceptum. Endoscopic treatment for JGI has been suggested in some case reports; however, this treatment is associated with a significant risk of recurrence [4, 5, 24].

In conclusion, JGI is a rare but potentially fatal complication after gastric surgery, especially following Whipple’s procedure. Early diagnosis and treatment are crucial, and surgery is considered the most effective treatment for JGI.

## Data Availability

Not applicable.

## Abbreviations

JGI: Jejunogastric intussusceptions
CT: Computed Tomography
US: Ultrasound
